# *In vitro* Type II Restriction of Bacteriophage DNA With Modified Pyrimidines

**DOI:** 10.3389/fmicb.2020.604618

**Published:** 2020-10-27

**Authors:** Kiersten Flodman, Ivan R. Corrêa, Nan Dai, Peter Weigele, Shuang-yong Xu

**Affiliations:** New England Biolabs, Inc., Ipswich, MA, United States

**Keywords:** Type II restriction, modified phage genome, phage SP8, phage Xp12 genome sequence, 5hmdU DNA kinase

## Abstract

To counteract host-encoded restriction systems, bacteriophages (phages) incorporate modified bases in their genomes. For example, phages carry in their genomes modified pyrimidines such as 5-hydroxymethyl-cytosine (5hmC) in T4*gt* deficient in α- and β-glycosyltransferases, glucosylated-5-hydroxymethylcytosine (5gmC) in T4, 5-methylcytosine (5mC) in Xp12, and 5-hydroxymethyldeoxyuridine (5hmdU) in SP8. In this work we sequenced phage Xp12 and SP8 genomes and examined Type II restriction of T4*gt*, T4, Xp12, and SP8 phage DNAs. T4*gt*, T4, and Xp12 genomes showed resistance to 81.9% (186 out of 227 enzymes tested), 94.3% (214 out of 227 enzymes tested), and 89.9% (196 out of 218 enzymes tested), respectively, commercially available Type II restriction endonucleases (REases). The SP8 genome, however, was resistant to only ∼8.3% of these enzymes (17 out of 204 enzymes tested). SP8 DNA could be further modified by adenine DNA methyltransferases (MTases) such as M.Dam and M.EcoGII as well as a number of cytosine DNA MTases, such as CpG methylase. The 5hmdU base in SP8 DNA was phosphorylated by treatment with a 5hmdU DNA kinase to achieve ∼20% phosphorylated 5hmdU, resulting resistance or partially resistant to more Type II restriction. This work provides a convenient reference for molecular biologists working with modified pyrimidines and using REases. The genomic sequences of phage Xp12 and SP8 lay the foundation for further studies on genetic pathways for 5mC and 5hmdU DNA base modifications and for comparative phage genomics.

## Introduction

Type II restriction and modification (R-M) systems encoded by bacteria and archaebacteria restrict foreign DNA from bacteriophages (phages) and mobile genetic elements (Smith, 1979). The companion methyltransferase (MTase) usually modifies the host genome rendering it refractory to the endonuclease and thereby preventing self-restriction (reviewed in Pingoud and Jeltsch, 2001; Pingoud et al., 2016). To counteract host restriction systems, many phages synthesize heavily modified (aka hypermodified) bases in their genomes by a combination of biosynthetic activities acting before and/or after DNA replication (Gold and Schweiger, 1969; Weigele and Raleigh, 2016; Lee et al., 2018). Additionally, some phage or prophage genomes encode multi-specificity C5 methyltransferases (Schumann et al., 1996; Xu et al., 2012), Dam methylase (Hattman et al., 1985), or frequent adenine MTase (Drozdz et al., 2012) to similarly protect phage genome from Type II restriction. Coliphage T4 synthesizes the hypermodified base, glucosylated 5-hydroxymethylcytosine completely replacing cytosine in its genomic DNA and as a result, can infect *Escherichia coli* (*E. coli*) strains encoding McrBC and McrA, which are modification-dependent restriction systems that attack 5hmC/5mC-containing DNA (Lehman and Pratt, 1960; Fleischman et al., 1976; Raleigh et al., 1989). Phage T4*gt* is a mutant deficient in DNA α- and β-glucosyltransferases and as a result contains 5-hydroxymethycytosines (5hmC) in its genome (Miller et al., 2003) and thus is restricted by the host McrBC and McrA systems. Phage Xp12 (natural host *Xanthomonas oryzae*) contains 5-methylcytosines replacing all cytosines in its genome (Kuo et al., 1968a,b). The *Bacillus* phage SP8, like the phages SPO1 and ϕe, contains 5-hydroxymethyl-2′-deoxyuridine (5hmdU) in its genome (Hoet et al., 1992).

It has been known for many years since the discovery of Type II R-M systems that these modified phage genomes are somewhat resistant to Type II restriction *in vitro* (Miller et al., 1985). But only limited information is available for a small number of restriction endonucleases (REases) on heavily modified phage genomes (Huang et al., 1982). The goal of this work is to test a vast array of commercially available REases (Roberts et al., 2015) on the four phage DNAs (T4*gt*, T4, Xp12, and SP8) and compile a reference list for molecular biologists who use REases to create recombinant DNA. As part of this goal, we sequenced phages Xp12 and SP8 genomes and deposited the sequences in GenBank. In addition, we confirmed the modified base compositions in these four phage genomes by LC-MS. We examined several adenine and cytosine methyltransferase activities on SP8 DNA. We also tested Type II restriction on SP8 DNA after its phosphorylation by treatment with 5hmdU DNA kinase. The results detailed herein comprise a comprehensive reference for the *in vitro* activity of Type II R-M enzymes on hypermodified DNA.

## Materials and Methods

### Phage DNA Purification and Restriction Digestions

Bacteriophages Xp12 and SP8 were obtained from ATCC (#35934-B1 and #15563-B1, respectively). Bacteriophages T4 and T4*gt* were kindly provided by Dr. Elisabeth Raleigh (NEB). T4GT7 DNA was provided by Dr. Geoff Wilson (NEB). The bacteriophages supplying the genomic DNAs used in this study were cultured by infection of host cells at early log phase in liquid medium/broth cultured until a significant drop in optical density (OD) occurred indicating lysis of the majority of the cells in the culture. Phage particles were precipitated from centrifugated clarified lysates by addition of PEG8000 to 10% weight-by-volume (w/v) and 1 M NaCl to the phage lysates and collected by centrifugation. Phages were further purified by cesium chloride density gradient centrifugation and dialyzed against three changes of phage buffer (50 mM Tris–HCl, pH 7.5, 75 mM NaCl, 10 mM MgCl_2_). DNA was extracted from the phage by phenol-CHCl_3_ extraction, and ethanol precipitation (Sambrook et al., 1989).

REases, MTases, 5hmdU DNA kinase, and Proteinase K were provided by New England Biolabs, Inc., (NEB). NEBcutter v2.1 software (Vincze et al., 2003) was used to generate restriction patterns of phage DNA with the assumption of no base modification. We used excess of REases in restriction digestions (5 to 40 U to cleave 0.25 to 0.5 μg phage DNA) in 50 μl total volume incubated at the recommended temperature for 1 h (e.g., 5 μl of REases for low concentration enzyme supplied at 1,000 U/ml, 2 μl of REase for high concentration REase supplied at 20,000 U/ml). Four general restriction buffers (NEBuffer 1.1 (low salt), 2.1 (medium salt), 3.1 (high salt) and CutSmart buffer were used except those unique buffers recommended by the enzyme supplier. Digested DNAs were analyzed by agarose gel electrophoresis (0.8–1% gel). The DNA cleavage patterns were compared to NEBcutter-generated restriction patterns to determine digestion results as complete (C), partial (P), very partial (VP), or resistant (X) to digestions. To test phosphorylation of 5hmdU base in SP8 viral DNA, the DNA was first treated with 5hmdU DNA kinase for 2 h at 37°C in the presence of ATP (1 mM) and subsequently purified by spin column purification (NEB Monarch DNA clean up kit) before being subjected to nucleoside analysis (see below).

### Methylation and Challenge With REases to Check Methylation Level

SP8 phage DNA was methylated by treatment with excess DNA MTase and methyl-donor SAM in the recommended buffer for 2 h. After heat inactivation of the MTase (65°C for 30 min), the methylated DNA was digested by cognate or non-cognate REases to evaluate the degree of resistance to restriction.

### Determination of DNA Base Compositions by Liquid Chromatography-Mass Spectrometry (LC-MS)

Modified or unmodified phage DNA was precipitated in ethanol, dried, and stored at −20°C. DNA samples (5 μg) were digested to nucleosides by treatment with the Nucleoside Digestion Mix (NEB, M0649S) overnight at 37°C. Nucleoside analysis was performed on an Agilent LC/MS System 1200 Series instrument equipped with a G1315D diode array detector and a 6120 Single Quadrupole Mass Detector operating in positive (+ESI) and negative (−ESI) electrospray ionization modes. LC was carried out on a Waters Atlantis T3 column (4.6 mm × 150 mm, 3 μm) with a gradient mobile phase consisting of 10 mM aqueous ammonium acetate (pH 4.5) and methanol. MS data acquisition was recorded in total ion chromatogram (TIC) mode. Each nucleoside was identified as follows: dC [M + H]^+^ 228.1 and [M − H]^–^ 226.2; dG [M + H]^+^ 268.1 and [M − H]^–^ 266.1; dT [M + H]^+^ 243.1 and [M − H]^–^ 241.1; dA [M + H]^+^ 252.1 and [M − H]^–^ 250.1; 5mdC [M + H]^+^ 242.1 and [M − H]^–^ 240.2; 6mdA [M + H]^+^ 266.1 and [M − H]^–^ 264.1; 5hmdC [M + H]^+^ 258.1 and [M − H]^–^ 256.1; α- and β-5hmdC [M + H]^+^ 420.2 and [M − H]^–^ 418.1. The relative abundance of each nucleoside was determined by dividing the UV absorbance by the corresponding extinction coefficient at 260 nm. To estimate the ratio of phosphorylation of 5-hydroxymethyluridine, kinase-treated SP8 gDNA was digested to nucleotides by treatment with Nuclease P1 (NEB, M0660S) overnight at 37°C. Each nucleotide was identified as follows: dCMP [M + H]^+^ 308.0 and [M − H]^–^ 306.0; 5hmdUMP [M − H]^–^ 337.0; dGMP [M + H]^+^ 348.0 and [M − H]^–^ 346.0; dAMP [M + H]^+^ 332.0 and [M − H]^–^ 330.0.

### Sequencing Xp12 and SP8 Phage Genomes

Samples of genomic DNA extracted from bacteriophages Xp12 and SP8 were sheared to ∼5 kb average fragment length using the Covaris gTube (Covaris Inc., Woburn, MA) according to manufacturer’s instructions. A total of 5 μg of sheared genomic DNA was used to prepare libraries for Pacific Biosciences (PacBio) Single Molecule Real-Time (SMRT) sequencing on the RSII model sequencer using P6-C4 chemistry and a flow-cell for each phage library. Following sequencing, reads were *de novo* assembled using the HGAP2 algorithm each yielding a single contig with average 200-fold coverage. Open reading frames and some gene assignments were performed by Rapid Annotation of Subsystems Technology (RAST) via web server^1^ (Aziz et al., 2008). The phage genome sequences for Xp12 and SP8 have been deposited in GenBank.

## Results

### Base Composition Analysis of Modified Phage Genomes

**FIGURE 1:**
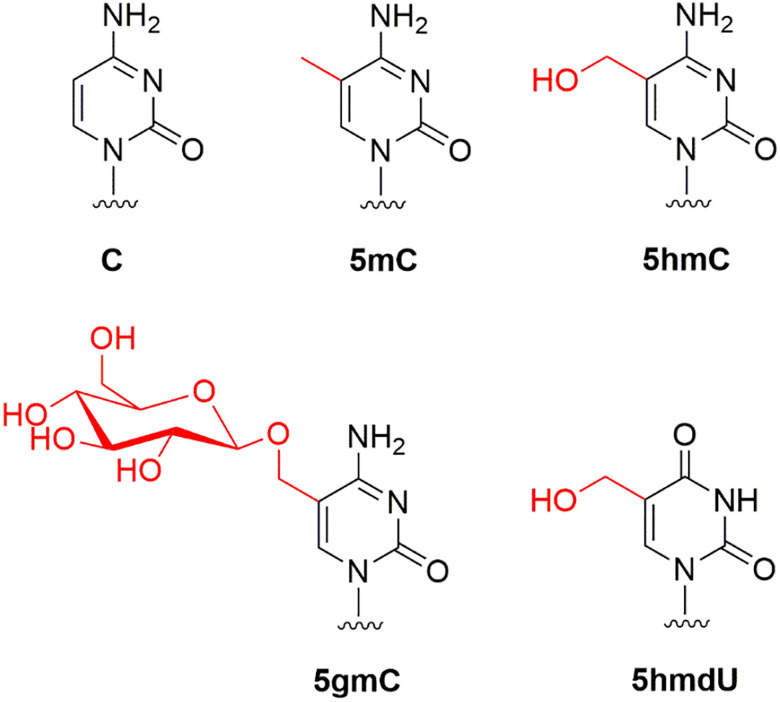
Modified bases found in phage T4*gt* (5hmC), T4 (5gmC), Xp12 (5mC), and SP8 (5hmdU).

Phage DNAs were extracted from phage particles, purified by the CsCl gradient centrifugation and had their base composition analyzed by the LC-MS as described previously (Flodman et al., 2019). Table 1 shows the base composition of seven phage genomes. T4GT7, a mutant deficient in 5hmC synthesis (containing only canonical cytosines in its genome) (Snyder et al., 1976), and phage λ were used as controls for host-encoded M.Dam and M.Dcm transient methylation. Figure 1 shows the modified bases 5mC, 5hmC, 5gmC, and 5hmdU found in four phage genomes.

**TABLE 1:** Percentage of modified cytosine, adenine and 5hmdU bases in phage genomes.

**Phage**	**C**	**5mC^*b*^**	**5hmC**	**5gmC**	**6mA^*c*^**	**5hmdU**
T4GT7^*a*^ (Dam^+^ Dcm^+^)	>99%	**0.3%**	None	None	**0.5%**	None
T4*gt*	None	None	**83%**	**10% α-5gmC 7% β-5gmC**	**0.5%**	None
T4	None	None	None	**60% α-5gmC 40% β-5gmC**	**0.4%**	None
Xp12	None	**100%**	None	None	None	None
SP8	>99%	None	None	None	None	**100%**
SP8 + 5hmdU DNA kinase	>99%	None	None	None	None	**80% 5hmdU 20%**
						**phosphorylated-5hmdU**
Lambda (Dam^+^ Dcm^+^)	>99%	**0.4%**	None	None	**0.7%**	None
Lambda (Dam^–^ Dcm^+^)	>99%	**0.4%**	None	None	<0.05%	None

*^a^Bacteriophage T4 GT7 [amC87(42^–^), amE51(56^–^), NB5060(Δ rllB^–^ denB^–^ ac), *unf* 39(*alc*)].*

*^*b*^Host Dcm (CCWGG) partially modified phage T4GT7 and Lambda.*

*^*c*^Host Dam and phage T4 GATC methyltransferase partially modified phage DNA.*

*Bold values indicate the percentage of base modification.*

### Type II Restriction of Phage T4*gt*, T4, Xp12 and SP8 Genomic DNA

In recent years, stable levels 5hmC were discovered in human and mouse stems cells and brain cells. This base derives from an active DNA demethylation pathway involving the oxidation of 5mC by Ten-eleven-translocation (TET; 5-methylcytosine dioxygenase) enzyme: 5mC, 5hmC, 5fC (5-formylcytosine), 5caC (5-carboxycytosine) and subsequent removal by the thymine DNA glycosylase (TDG) repair enzyme (Tahiliani et al., 2009; He et al., 2011; Parker et al., 2019). Thus, there is a practical need in knowing how REases perform on 5mC or 5hmC modified DNA. We examined Type II restriction enzyme activity (enzymes commercially available from NEB) on four modified phage gDNAs. Phage DNA sensitivity or resistance to Type II restriction is summarized in Table 1, and all restriction data is presented in Supplementary Tables S1–S4. T4*gt*, T4, and Xp12 genome show 81.9, 94.3, and 89.9% resistance to all Type II REases, respectively (Table 1 and Supplementary Figure S1. See below). The SP8 genome (5hmdU), however, was resistant to only ∼8.3% of all Type II restrictions. The 5hmdU bases can be further modified as in phages ViI and ΦW-14 to provide higher resistance (Flodman et al., 2019). In addition, a simple phosphorylation step by treatment with 5hmdU DNA kinase can also increase DNA resistance to Type II restriction (see below).

### Restriction of Phage T4*gt* DNA

**FIGURE 2:**
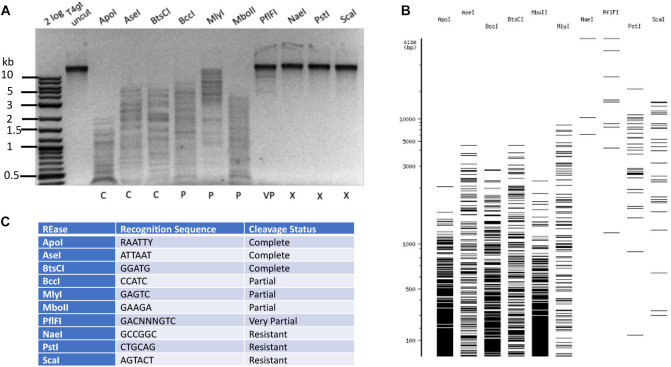
Type II restriction of phage T4*gt* DNA. Phage T4*gt* gDNA was digested by 10 REases and analyzed by agarose gel electrophoresis **(A)**. 2 log, DNA size marker in 100 bp to 10 kb (NEB). The predicted digestion patterns by NEBcutter are shown in **(B)**. The target recognition sequence and digestion results are summarized in **(C)**. C = complete digestion, P = partial digestion, VP = very partial digestion (only a few weak bands), X = resistant to restriction.

The DNA of phage T4*gt*, a mutant T4 strain having diminished glucosyltransferase activity, contains 5hmC replacing all C in its genome. Due to the presence of 5hmC, the gDNA was previously reported by others to be resistant to some restriction digestions *in vitro* (NEB catalog 2019/20), but an extensive list of all commercially available REases is lacking. We purified phage T4*gt* DNA and analyzed its base composition. T4*gt* genomic DNA contained ∼83% of 5hmC, and 10% of α-5gmC and 7% of β-5gmC among all cytosine bases (Supplementary Figure S2A). The residual amount of 5gmC is probably a result of low activity of the mutated α- and β-glucosyltransferases. Null mutant in α- and β-glucosyltransferase genes would be required to completely substitute all 5gmC bases with 5hmC. A small amount of modified adenine 6mA (0.5%) was also detected in the T4*gt* DNA, which may originate from either the host Dam or phage-encoded Dam methyltransferase (AF158101). T4*gt* DNA can also be used as a substrate for modification-dependent endonucleases (see below). Despite the presence of residual 5gmC, we decided to use the T4*gt* DNA for testing Type II restriction. Examples of restriction digestion are shown in Figure 2. *Apo*I (RAATTY), *Ase*I (ATTAAT), and a Type IIS enzyme BtsCI (GGATGN2↓) completely digested the DNA. The DNA is nearly resistant to digestion by *Pfl*FI (GACN3GTC), and completely resistant to digestion by *Nae*I (GCCGGC), *Pst*I (CTGCAG), or *Sca*I (AGTACT). While *Bcc*I (CCATC), *Mly*I (GAGTC), and *Mbo*II (GAAGA) partially digested the substrate. Further restriction results are presented in Supplementary Table S1. Overall, phage T4*gt* was resistant to 81.9% of all Type II restriction enzymes tested here (186 out of 227 enzymes).

### Restriction of Phage T4 DNA

**FIGURE 3:**
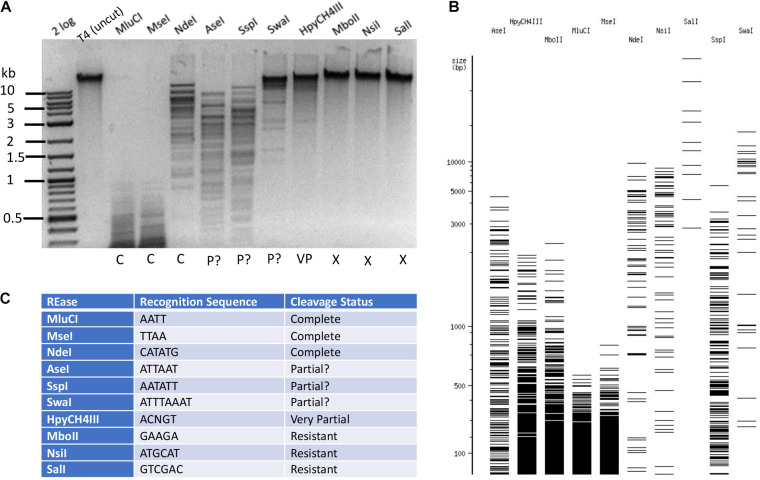
Type II restriction of phage T4 DNA. Phage T4 DNA was digested by 10 REases and analyzed by agarose gel electrophoresis **(A)**. The predicted digestion patterns in the absence of modifications by NEBcutter are shown in **(B)**. The recognition sequence and results are shown in **(C)**. The question marks in “P?” indicate possible partial digestions (the presence of a few large DNA bands not seen in NEBcutter patterns).

The base composition of phage T4 DNA was confirmed by LC-MS analysis to carry 60% α-5gmC and 40% β-5gmC (Supplementary Figure S2B). Based on cleavage patterns obtained for phage T4*gt* DNA, it is reasonable to assume that negative restriction on T4*gt* would be mirrored on T4 DNA. To confirm this hypothesis, we tested twenty enzymes and found that all of them were negative on both T4*gt* and T4 (data not shown). Next, we selected and tested a subset of REases that could either completely or partially cleave T4*gt*. Examples of Type II restriction of phage T4 DNA are shown in Figure 3. MluCI (AATT), *Mse*I (TTAA), and *Nde*I (CATATG) digested T4 DNA completely. But *Ase*I (ATTAAT), *Ssp*I (AATATT), and *Swa*I (ATTTAAAT) did not show complete digestion, even though they all recognize sites containing A/T sequence and they do not overlap with *dam* methylation site. This partial digestion was reproducible (data not shown) and suggested an indirect inhibitory effect of 5gmC modifications on REases having only A/T nucleotides in their targets. T4 DNA was nearly resistant to *Hpy*CH4III (ACNGT) restriction, and completely resistant to restriction by *Mbo*II (GAAGA), *Nsi*I (ATGCAT), or *Sal*I (GTCGAC), all of which containing 2–4 modified cytosines in their target sites. Further Type II restriction data are presented in Supplementary Table S2. Phage T4 DNA was resistant to 94.3% of all Type II restrictions examined here (214 out of 227 enzymes), indicating the highly resistant nature of 5gmC modified DNA. In the arms race between phage and host, bacteria had developed modification-dependent restriction systems to restrict such hypermodified genomes (see below).

Hydroxymethylation-deficient T4 GT7 and λ DNA were used as controls. Only low levels of modified bases 5mC (0.3%) and 6mA (0.5%) were found in phage T4 GT7 (Supplementary Figure S3). The 5mC and 6mA bases are most likely transiently modified by the bacterial host enzymes M.Dcm and M.Dam. Similarly, low levels of modified bases 5mC and 6mA were found in Dcm^+^ Dam^+^ phage λ DNA (Supplementary Figure S4A). As could be expected, Dam^–^ phage λ DNA displayed a much lower level of 6mA (<0.05%) (Supplementary Figure S4B). Type II restriction data of phage T4 GT7 DNA are presented in Supplementary Table S4 (spread sheet in the Supplementary).

### Restriction of Phage Xp12 DNA

**FIGURE 4:**
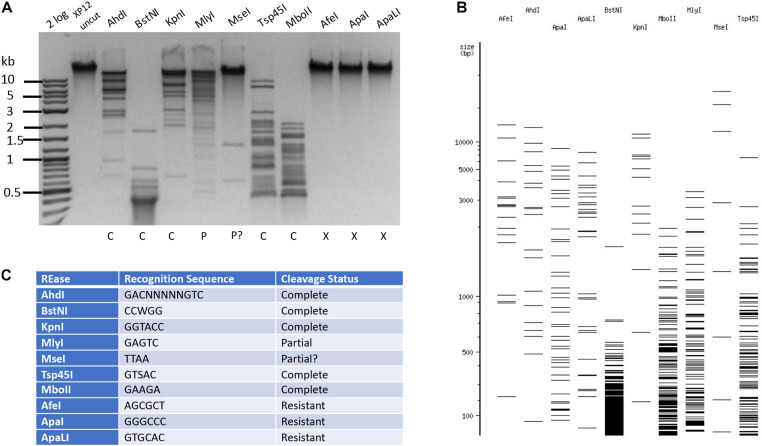
Restriction of phage Xp12 DNA. Example of 10 restriction digestions analyzed in 1% agarose gel **(A)**. NEBcutter predicted cleavage patterns in the absence of cytosine modification **(B)**. Enzyme recognition sequences and cleavage result **(C)**.

The phage Xp12 genomic DNA was sequenced using PacBio sequencing kit. The viral genome contains 63,783 bp (GenBank accession number MT664984). The detailed analysis of the Xp12 genome and 5mC modification pathway will be published elsewhere (PW). Examples of Type II RE cleavage of phage Xp12 DNA (5mC) are shown in Figure 4. The DNA was resistant to restrictions by *Afe*I (AGCGCT), *Apa*I (GGGCCC), and *Apa*LI (GTGCAC). It was partially resistant to Type IIS enzymes *Mly*I (GAGTC) and *Mse*I (TTAA) (a 3 kb *Mse*I fragment was missing). *Ahd*I, *Bst*NI, *Kpn*I, *Mbo*II, and TspA15I completely digested Xp12 DNA, even though their target sites contain 2–5 modified cytosines. It is known that M. *Ahd*I, M. *Kpn*I, and M1. *Mbo*II (GGAGG) are N6-adenine MTases and M2. *Mbo*II (TCTTC) is an N4-cytosine MTase (Roberts et al., 2015). The non-cognate C5 methylations did not have inhibitory effect on these REases. Further Type II restriction data are presented in Supplementary Table S3. Phage Xp12 DNA was nearly resistant to 90% of all Type II restrictions tested here (196 out of 218 enzymes).

### Restriction of Phage SP8 DNA

**FIGURE 5:**
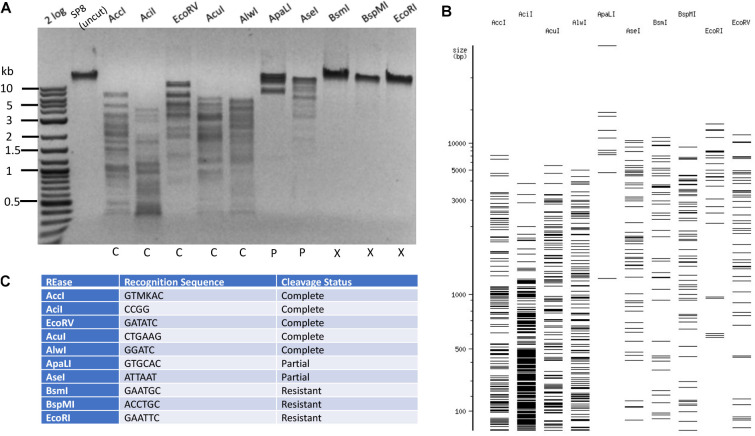
Restriction of phage SP8 (5hmdU) DNA. Example of 10 restriction digestions analyzed in 1% agarose gel **(A)**. NEBcutter predicted cleavage patterns in the absence of T modification **(B)**. Enzyme recognition sequences and restriction result **(C)**.

The phage SP8 genomic DNA was sequenced by PacBio sequencing platform. The viral genome contains 138,741 bp (GenBank accession number MW001214). The *Bacillus* phage SP8 contains 100% 5hmdU replacing all T in its genome (Hoet et al., 1992). LC-MS analysis of SP8 DNA confirmed its predicted base composition (Table 1 and Supplementary Figure S5). In the time since the publication of a study examining cleavage of 5hmdU DNA by a small set of REases (Huang et al., 1982), many more Type II REases have become commercially available. We decided to test all currently available Type II REases on SP8 DNA. Examples of Type II restriction of phage SP8 DNA are shown in Figure 5. Phage SP8 DNA was partially resistant to restriction by *Apa*LI (GTGCAC) and *Ase*I (ATTAAT), and completely resistant to restriction by *Bsm*I (GAATGC with three 5hmdU), *Bsp*MI (ACCTGC), and *Eco*RI (GAATTC). Five REases (*Acc*I, *Aci*I, AcuI, *Alw*I, and *Eco*RV) completely digested the substrate DNA. While both *Eco*RI and *Eco*RV sites contain four 5hmdU replacing T in SP8 DNA, its sensitivity to restriction was completely different: SP8 DNA was resistant to *Eco*RI digestion (24 *Eco*RI sites in the genome) but sensitive to *Eco*RV restriction. The genomic DNA can be digested by MluCI (AATT). Therefore, it is difficult to predict which REase will completely digest SP8 DNA, even with the presence of up to six 5hmdUs in their target recognition sequences. The SP8 genome was resistant to only a small fraction (∼8.3%) of all Type II restrictions tested here (17 out of 204 enzymes, Supplementary Table S5). Type II restrictions of four phage genomes are summarized in Table 2.

**TABLE 2:** Summary of Type II restrictions on phage T4*gt*, T4, Xp12, and SP8 gDNA.

**Cleavage Status**	**T4*gt***	**T4**	**Xp12**	**SP8**
Complete (C)	7.9%	4%	5.5%	67.2%
Partial (P)	7.9%	1.8%	3.2%	15.2%
Very Partial (VP)^*a*^	4.8%	0.9%	4.1%	3.9%
Resistant (X)	77.1%	93.4%	85.8%	4.4%
(VP + Resistant)	(81.9%)	(94.3%)	(89.9%)	(8.3%)
Inconclusive^*b*^	2.2%	0%	1.4%	9.3%

*^*a*^Very partial, only a few weak bands were visible in the agarose gel. ^*b*^Restriction fragment(s) larger than 10 kb were not clearly resolved in 1% agarose gel.*

### *In vitro* Methylation of 5hmdU DNA

Next, we tested a number of DNA methyltransferases on phage SP8 gDNA to see whether 5hmdU could potentially interfere with adenine methylation. After DNA methylation, the modified DNA was subjected to either cognate or non-cognate restriction enzymes. SP8 DNA was partially modified by M.Dam (GATC) or M.EcoGII (frequent adenine methyltransferase). The modified DNA became partially resistant to restriction by *Mbo*I (GATC, restriction blocked by 6mA modification). SP8 DNA, however, was a poor substrate for M. *Taq*I (Supplementary Figure S5). In a control experiment, M. *Taq*I was able to modify λ DNA and rendered the DNA resistant to *Taq*I restriction (data not shown). M. *Eco*RI was able to modify λ DNA and protect it from *Eco*RI restriction. However, in the case of SP8 DNA, no conclusion was derived from the results because the substrate DNA itself was already resistant to *Eco*RI even before methylation (24 *Eco*RI sites present in the genome). We have not analyzed other adenine methyltransferase activities on phage SP8 DNA due to the lack of enzyme availability. Overall, phage SP8 DNA can be efficiently methylated by C5 MTases (M. *Hae*III, M. *Msp*I, M. *Hha*I, M. *Alu*I, M. *Hpa*II, CpG, and GpC methyltransferases) as one expected. These MTases could modify phage SP8 efficiently and render the DNA resistant to both cognate restriction and overlapping restriction (Supplementary Figure S6). The methylation results are summarized in Table 3.

**TABLE 3:** DNA methylation by adenine or cytosine MTases followed by restriction challenge of the methylated phage SP8 DNA.

**Type of DNA MTase**	**Sequence modified**	**REase used to challenge DNA**	**Modification status**	**Cleavage of gDNA prior to modification**
**6mA MTase**				
M. *Taq*I	TCGA	*Taq*I	Very Partial	Complete (*Taq*I)
M.Dam	GATC	*Mbo*I	Partial	Complete (*Mbo*I)
M.EcoGII	A	*Mbo*I	Partial	Complete (*Mbo*I)
**5mC MTase**				
M. *Hae*III	GGCC	*Hae*III	Complete	Complete
M. *Msp*I	CCGG	*Msp*I	Complete	Complete
M. *Hha*I	GCGC	*Hha*I	Complete	Complete
M. *Alu*I	AGCT	*Alu*I	Complete	Complete
CpG	CG	*Hpa*II	Complete	Complete
M. *Hpa*II	CCGG	*Hpa*II	Complete	Complete
GpC	GC	*Hha*I	Complete	Complete

### Modification-Dependent Restriction Endonuclease (MDRE) Activity on T4*gt*, T4, Xp12, and T4GT7 DNA

MDREs (Type IIM and Type IV) cleave modified DNA specifically (Roberts et al., 2003). These enzymes are thought to be evolved in bacterial hosts to attack modified phage genomes in the host-virus arms race (Bair and Black, 2007). We examined a few commercially available enzymes for activity on modified phage DNA. T4*gt*, T4, Xp12, and T4GT7 were digested with AbaSI, FspEI, LpnPI, MspJI, and McrBC. Table 4 summarizes the restriction results. All five enzymes digested T4*gt* DNA efficiently. Only AbaSI (a PvuRts1I homolog) was able to digest T4 and T4*gt* DNA (Wang et al., 2011). T4 DNA was resistant to restriction by FspEI, LpnPI, MspJI, and McrBC. The T4GT7 DNA was partially digested by FspEI and MspJI, probably due to partial M.Dcm methylation of the genomic DNA. AbaSI and McrBC were incapable of cleaving T4GT7 DNA. As expected, AbaSI was able to cleave 5hmC and 5gmC modified DNA, but not 5mC or unmodified DNA. FspEI, MspJI, and McrBC were able to cleave 5mC and 5hmC modified DNA, but not on 5gmC and unmodified DNA. It has been reported that GmrSD endonuclease is able to cleave both T4*gt* and T4 DNA in ATP/GTP dependent manner (He et al., 2015).

**TABLE 4:** Digestion of modified phage gDNA with MDREs.

	**AbaSI (5hmCN_20_G) (5gmCN_20_G)**	**FspEI (C5mC) (C5hmC)**	**LpnPI (C5mCDG) (C5hmCDG) D = not C**	**MspJI (5mCNNR) (5hmCNNR) R = A or G**	McrBC* (R5mC) (R5hmC)
**T4*gt***	+ ++	++	++	+ ++	+++
**T4**	+ ++	−	−	−	−
**Xp12**	±	+ +	++	++	± ?
**T4GT7**	−	+	±	+	−

*+ + +, positive, complete digestion. + +, positive, nearly complete digestion. +, positive, partial digestion. ±, slight smearing. ± ?, inconclusive (low activity). −, negative, no activity. ^∗^McrBC requires two sites separated by certain distance (55–3000 bp) and GTP hydrolysis for endonuclease activity.*

### 5hmdU DNA Kinase Activity: Phosphorylation of Phage SP8 DNA to Become More Resistant to Type II Restrictions

5hmdU DNA kinase can phosphorylate the 5-hydroxymethyl group in the 5hmdU in a sequence-specific manner (Lee et al., 2018) making the base more negatively charged. The 5hmdU DNA kinase has been shown to block *Nco*I restriction after the kinase reaction^2^. We used the 5hmdU DNA kinase to phosphorylate phage SP8 DNA. After the kinase treatment, the DNA was purified by spin column and its base composition was analyzed by LC-MS. Supplementary Figure S7 shows that ∼20% of 5hmdU had been converted to its phosphorylated form in SP8 DNA.

**FIGURE 6:**
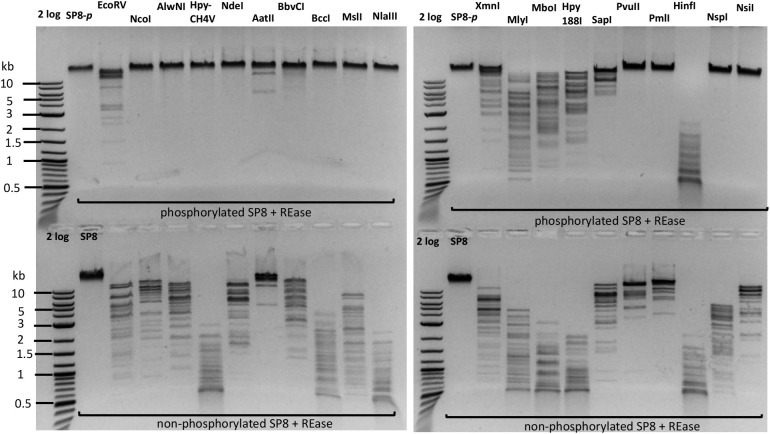
Restriction digestion of SP8 gDNA following treatment with 5hmdU DNA kinase. Top panel, restriction of phosphorylated SP8 DNA (SP8-*p*). Bottom panel, control: restriction of non-*p* phage SP8 DNA. The phosphorylated DNA appeared largely resistant to restrictions by *Nco*I (CCATGG), *Alw*NI (CAGN_3_CTG), *Hpy*CH4V (ACGTn), *Nde*I (CATATG), *Bbv*CI (CCTCAGC), *Bcc*I (CTATC), *Msl*I (CAYN_4_RTG), *Nla*III (CATG), *Pvu*II (CAGCTG), *Pml*I (CACGTG), *Nsp*I (RCATGY), and *Nsi*I (ATGCATn) digestion; and partially resistant to *Eco*RV (nGATATCn), *Mly*I (nGAGTCn), *Mbo*I (nGATCn), *Hpy*188I (TCNGA), *Sap*I (nGCTCTTC) digestion. Phosphorylation of 5hmdU had no inhibitory effect on *Aat*II (nGACGTC) and *Hin*fI (nGANTC) digestions. The TG, TC, nG, and Tn dinucleotides in the restriction sites are underlined.

Next, we set out to test more Type II restriction after phosphorylation. Phosphorylated phage SP8 DNA was tested with 20 restriction enzymes. Phosphorylated SP8 DNA was resistant to restriction by *Nco*I, *Alw*NI, *Nde*I, *Bbv*CI, *Bcc*I, *Msl*I, *Nla*III, *Pvu*II, *Pml*I, *Nsp*I, and *Nsi*I, and partially resistant to restriction by *Eco*RV, *Xmn*I, *Mly*I, *Mbo*I, and *Hpy*188I (Figure 6). Inspection of the resistant and partially resistant sites indicated that these sites contain TG (or NG) and TC dinucleotides in their recognition sequences, confirming previous findings with phage M6, ViI, and ϕW-14 DNAs (Flodman et al., 2019). This type of base modification (phosphorylation) is a useful method to render the site resistant to Type II restriction when a DNA MTase is not yet available to modify the same sequence.

## Discussion

### Type II Restriction of Modified Phage DNAs

In this work, we analyzed Type II restriction of modified phage genomes T4*gt* (5hmC), T4 (5gmC), Xp12 (5mC), and SP8 (5hmdU). We found T4*gt*, T4, and Xp12 genomes are highly resistant to Type II restriction; while SP8 DNA is only modestly resistant to Type II restriction.

A few REases that recognize only A/T target sites partially digested phage T4 and Xp12 DNA. It is not clear whether this observation is an artifact or true indirect effect of base modification on neighboring sequences. In any event, practitioners using REases to digest hypermodified phage genomes should be aware of this partial inhibition.

### Restriction of Modified Phage DNAs by MDREs

The MDREs (AbaSI, FspEI, LpnPI, MspJI, and McrBC) tested here digested phage T4*gt* DNA efficiently. No undigested phage DNA was detected. It is important to note that LC-MS analysis of T4*gt* DNA indicated that only 83% of cytosines are modified as 5hmC, with the remaining cytosines found in the form of 5gmC. We have observed a slight variation in phage T4*gt* base composition (83 to 90% of 5hmC vs. 10 to 17% of 5gmC) across independent batches of phage lysates. The reason for this variation is unknown, but it may be related to suppressor efficiency of different *E. coli* strains growing T4*gt* α- and β- glucosyltransferase amber mutants.

Phage T4 DNA shows highest resistance to bacteria-encoded Type II restriction. However, bacteria also evolved new restriction systems that can target 5gmC-modified DNA. Examples of these restriction systems include PvuRts1I-like enzymes (Janosi et al., 1994), GmrSD-like enzymes (He et al., 2015), and EVE-HNH endonucleases (Lutz et al., 2019). Many more Type IV restriction systems have been found to restrict 5mC and 5hmC modified DNA (Loenen and Raleigh, 2014).

### Methylation of Phage SP8 DNA and Phosphorylation of SP8 DNA

We demonstrated here that M.Dam and M.EcoGII could efficiently modify phage SP8 DNA. However, M. *Taq*I modified the viral DNA poorly. The cytosine MTases tested here mostly recognize G/C sequence (except M. *Alu*I) and they methylated SP8 DNA efficiently, rendering it resistant to cognate restriction or overlapping restriction. In addition, we tested Type II restriction of phosphorylated (*p*) SP8 DNA following treatment with 5hmdU DNA kinase. The kinase-treated DNA became more resistant to Type II restriction only if the targets sites contained TG (NG) or TC dinucleotides. A few enzymes (*Aat*II and *Hin*fI) were not affected by base phosphorylation. 5hmdU-containing plasmid or phage DNA could be potentially grown in an engineered *E. coli* strain to achieve ∼75% maximum incorporation (Mehta et al., 2016). Alternatively, PCR amplification could be used to incorporate 5hmdU using 5hmdUTP in the dNTP pool, depending on the efficiency of 5hmdUTP incorporation by a PCR DNA polymerase. We anticipate that 5hmdU DNA kinase will be a useful tool to manipulate DNA, making it resistant to certain restriction digestions, especially where DNA MTases are not commercially available.

### Photocaged DNA With dUMP and dCMP Derivatives

In addition to DNA methylation and 5hmdU phosphorylation, researchers also utilize dUMP derivatives for photocaging which can be reversed by UV and light treatment. For example, 5-[(2-Nitrobenzyl)oxymethyl]-2′-deoxyuridine 5′-*O*-triphosphate can be incorporated into DNA in PCR reaction and such photocaged DNA has been shown to be resistant to cleavage by *Afl*II (CTTAAG), *Kpn*I (GGTACC), *Pvu*II (CAGCTG), and *Rsa*I (GTAC) endonucleases. Deprotection of the photocaged DNA by UV light treatment (355–425 nm) converted it to 5hmdU-containing DNA which could be cleaved by the REases (Vanikova and Hocek, 2014; Bohacova et al., 2018a). Our work confirmed that phage SP8 DNA with natural 5hmdU modified bases can be cleaved by the four REases mentioned above. It has been shown that 5hmdU phosphorylation of PCR DNA also negatively impacted *in vitro* transcription efficiency by *E. coli* RNA polymerase and restriction by *Alu*I (AGCT) and *Rsa*I (GTAC) (Vanikova et al., 2019). *Alu*I site contains TN dinucleotides in both strands. But *Rsa*I site does not contain TG or TC dinucleotides. How the 5hmdU DNA kinase is able to phosphorylate the *Rsa*I site is not clear. Similarly, photocaged dCTP derivatives (dC^NB^TP and dC^NP^TP, NB, 2-nitrobenzyl; NP, 2-nitropiperonyl) can be incorporated into PCR DNA, which becomes resistant to *Rsa*I restriction. Photochemical release of the protected bases by UV or visible light treatment converted it to 5hmC-containing DNA, which is sensitive to *Rsa*I restriction (Bohacova et al., 2018b). We found that *Rsa*I partially digested T4*gt* DNA. The variation of *Rsa*I digestion results may result from the high density of 5hmC modification in the phage DNA vs. the synthetic PCR DNA described by others. The photocaged nucleotides provided a convenient and reversible way to control enzyme activities on modified DNA *in vitro*.

### GenBank Accession Number

Phage Xp12 and SP8 genome sequences have been deposited in GenBank and assigned the accession numbers MT664984 and MW001214.

## Data Availability Statement

The datasets presented in this study can be found in online repositories. The names of the repository/repositories and accession number(s) can be found below: https://www.ncbi.nlm.nih.gov/genbank/, MT664984 and MW001214.

## Author Contributions

KF, S-yX, IC, PW, and ND generated and analyzed experimental data. S-yX, KF, IC, and PW wrote the manuscript. All authors contributed to the article and approved the submitted version.

## Conflict of Interest

S-yX, IC, ND, and PW are employed by New England Biolabs, Inc. The remaining author declares that the research was conducted in the absence of any commercial or financial relationships that could be construed as a potential conflict of interest.

## Footnotes

1 https://rast.nmpdr.org/

2 www.neb.com
