# Supplementary material for: In vitro Type II Restriction of Bacteriophage DNA With Modified Pyrimidines
Source: Front Microbiol. 2020 Oct 27;11:604618. doi: 10.3389/fmicb.2020.604618 (PMC7653180; doi:10.3389/fmicb.2020.604618)

Supplement Materials
Suppl. Figures S1-S7
Suppl. Tables 1-4 (spreadsheet file)

In vitro Type II restriction of bacteriophage DNA with modified pyrimidines

Kiersten Flodman, Ivan R. Corrêa, Jr., Nan Dai, Peter Weigele*, and Shuang-yong Xu*

New England Biolabs, Inc. 240 County Road, Ipswich, MA 01938, USA

*Corresponding authors:
xus@neb.com
1-978-380-7287

Weigele@neb.com
1-978-380-7304

Running title: Type II restriction of modified phage DNA

Key words: Type II restriction, modified phage genome, phage SP8, phage Xp12 genome sequence, 5hmdU DNA kinase

Suppl. Fig. S1. Type II restriction of T4*gt*, T4, Xp12, and SP8 gDNA shown in pie chart format (same data as presented in Table 1). Phages T4*gt*, T4, and Xp12 genomes show resistance to 81.9%, 94.3%, and 89.9% of commercially available Type II restriction endonucleases (REases from NEB), respectively. The phage SP8 genome, however, is resistant to only ~8.3% of Type II restriction. Many Type II REases (67.2%) can digest phage SP8 DNA completely. Phage Xp12 and SP8 genome sequences have been deposited in GenBank and assigned the accession numbers MT664984 and MW001214.

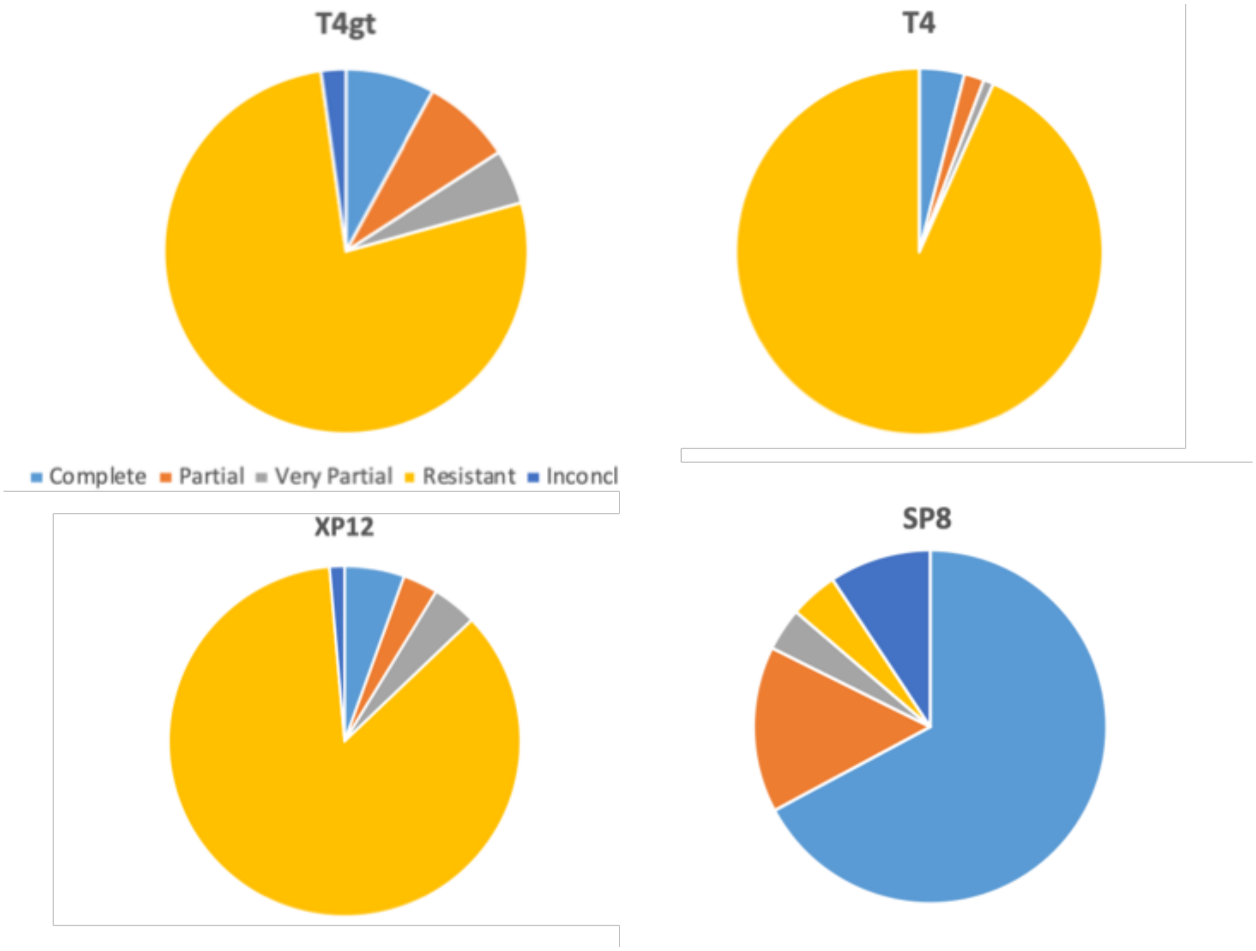

Suppl. Fig. S2. Base composition analysis of T4*gt* and T4 DNA. (**A**) The distribution of cytosine-derived bases 5hmC, α-5gmC and β-5gmC in T4*gt* was estimated by LC-MS at 83%, 10% and 7%, respectively. (**B**) The distribution of cytosine-derived bases α-5gmC and β-5gmC in T4 phage was estimated at 60% and 40%, respectively. The percentage of 6mA was estimated at 0.5% and 0.4% of the total adenosine nucleosides for T4*gt* and T4 gDNA, respectively.

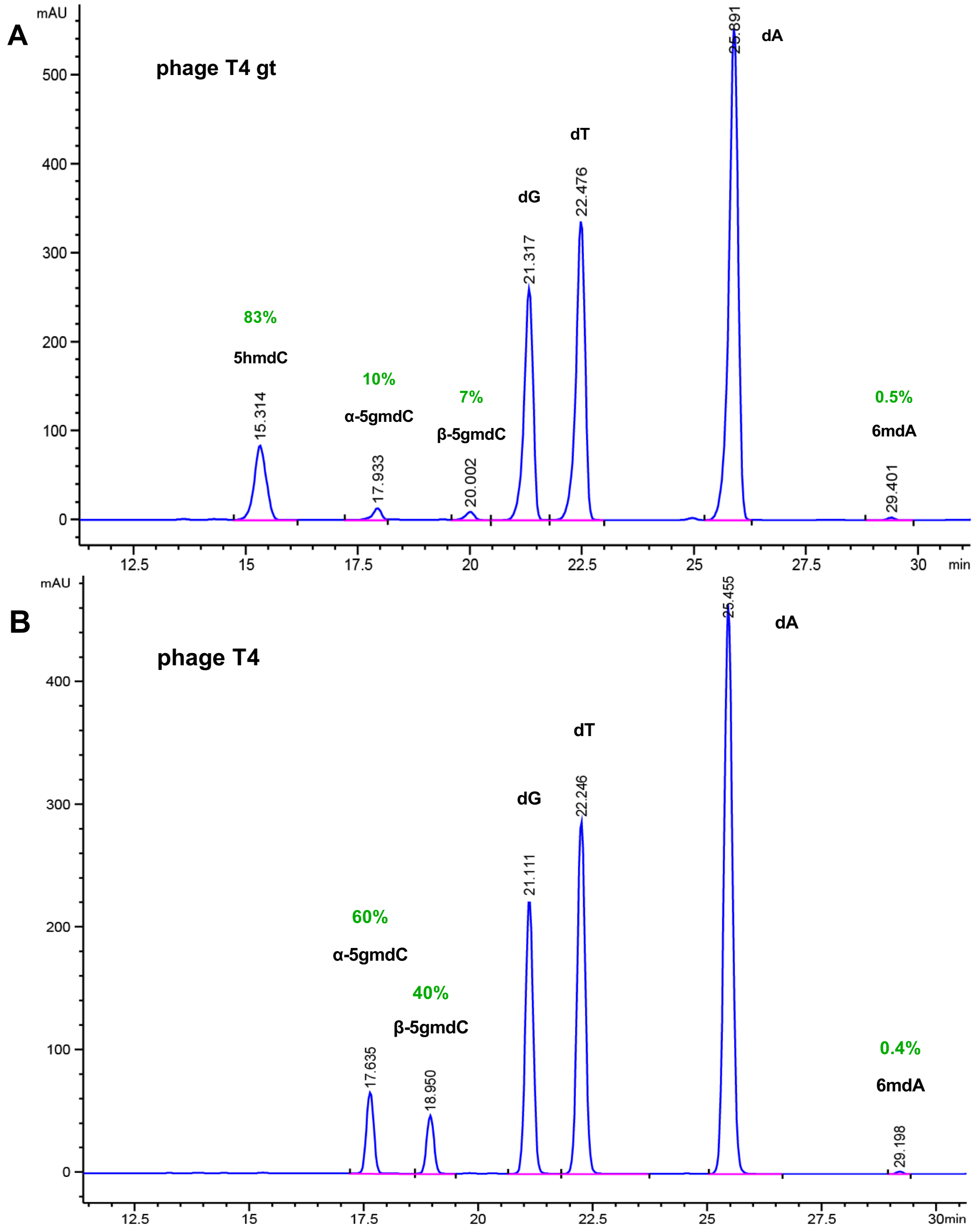

Suppl. Fig. S3. Base composition analysis of phage T4 GT7 DNA (unmodified control DNA). The percentages of 5mC and 6mA were estimated by LC-MS at 0.3% and 0.5% of the total cytidine and adenosine nucleosides, respectively. The modified bases 5mC (0.3%) and 6mA (0.5%) are presumably derived from transient methylation by host Dcm and Dam methylases or phage-encoded Dam methylase (phi.MTase).

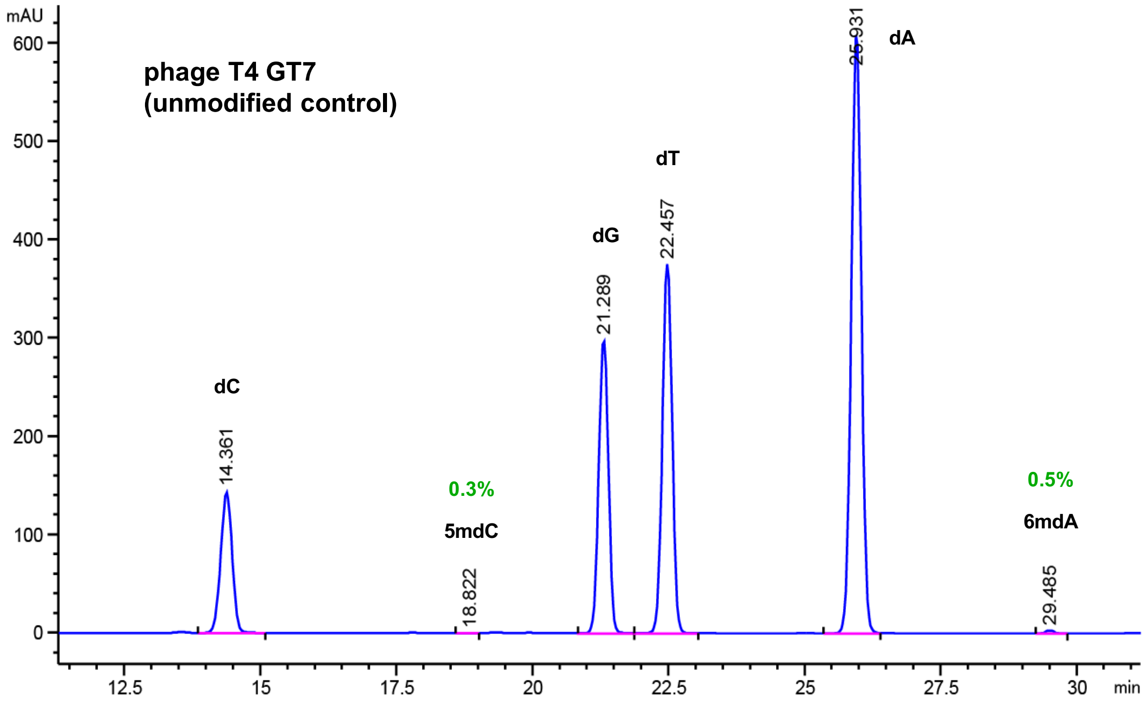

Suppl. Fig. S4. Base composition analysis of phage λ DNA. (**A**) The percentages of 5mC and 6mA in phage λ (Dcm^+ Dam^+) were estimated by LC-MS at 0.4% and 0.7% of the total cytidine and adenosine nucleosides, respectively. (**B**) The percentages of 5mC and 6mA in Dam-deficient phage λ were estimated by LC-MS at 0.4% and less than 0.05% of the total cytidine and adenosine nucleosides, respectively.

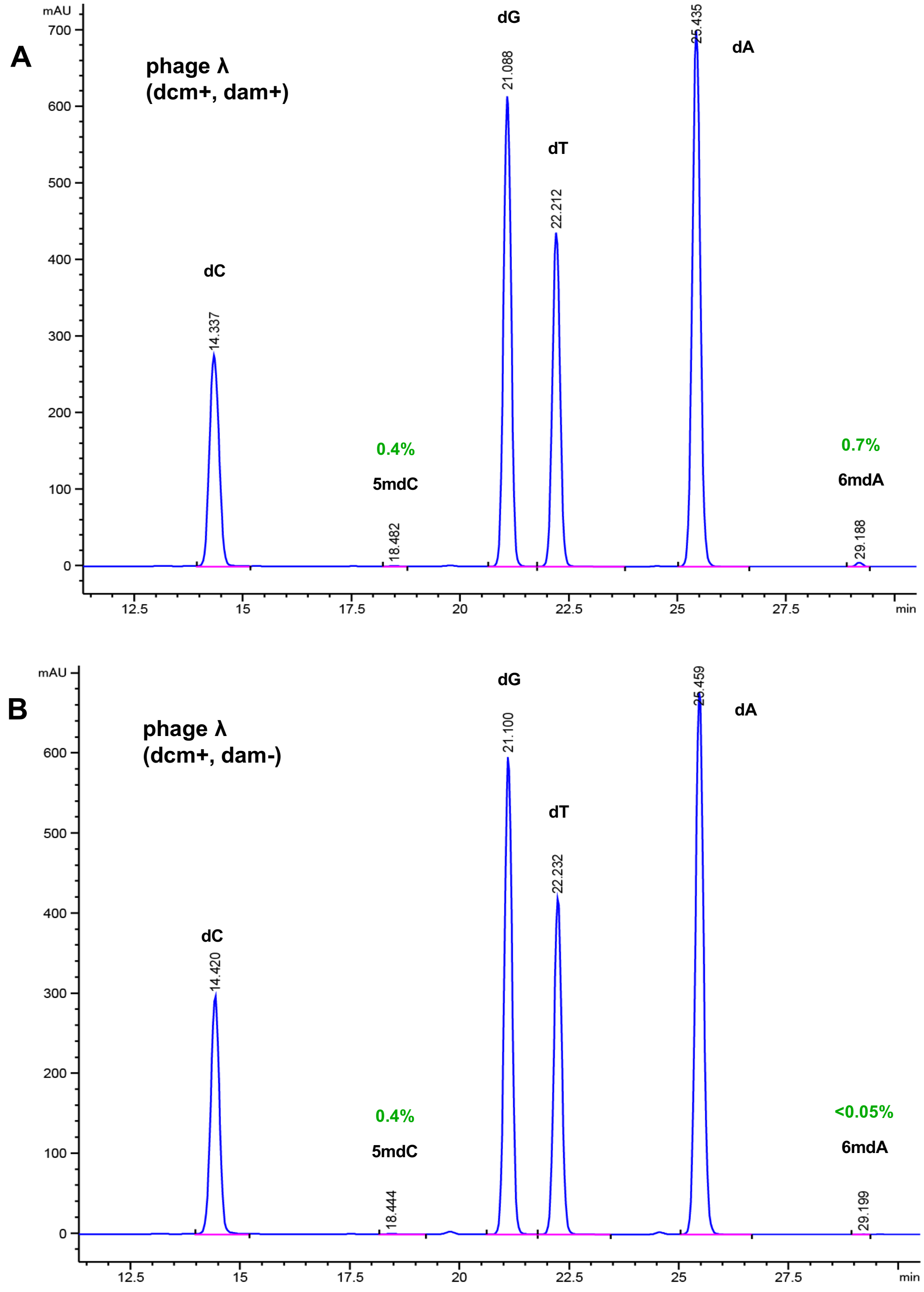

Suppl. Fig. S5. Adenine methylation of phage SP8 DNA.

Phage SP8 DNA was treated with M.EcoRI, M.TaqI, Dam methylase, and M.EcoGII, respectively, and the modified DNA was subsequently challenged with EcoRI, TaqI, or MboI REases and analyzed by agarose gel electrophoresis.

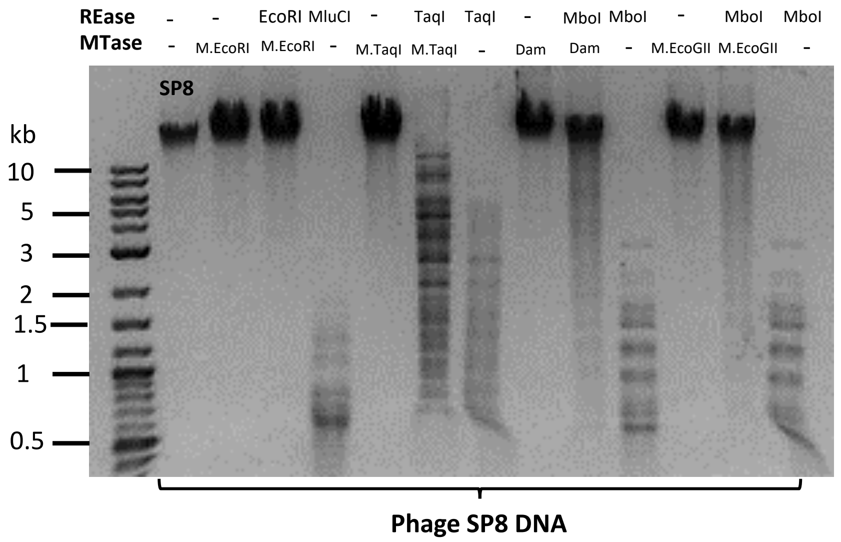

Suppl. Fig. S6. Cytosine methylation of phage SP8 DNA and subsequent challenge with REases. Phage SP8 DNA methylated by M.HaeIII, M.MspI, M.HhaI, M.AluI, M.HpaII, CpG methylase (M.SssI) and GpC methylase, respectively, and subsequently digested by the cognate or non-cognate restriction enzyme.

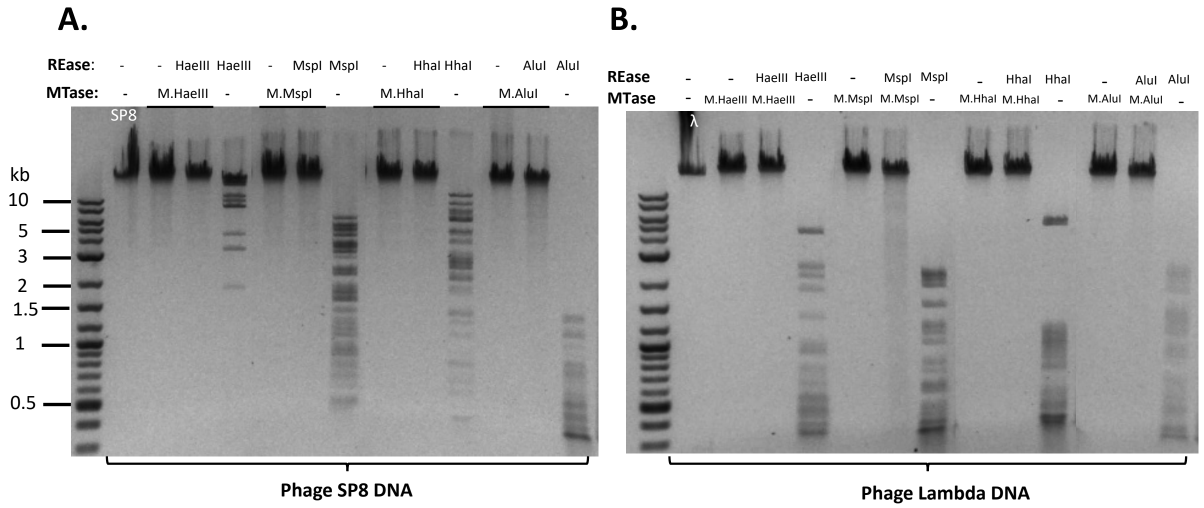

Suppl. Fig. S7. Base composition analysis of phage SP8 DNA. (**A**) Untreated SP8 gDNA was digested to nucleosides. LC-MS analysis indicated 100% replacement of dT with 5hmdU. (**B**) Phage SP8 after treatment with 5hmdU DNA kinase. To estimate the ratio of phosphorylation (*p*) of 5-hydroxymethyluridine, kinase-treated SP8 DNA was digested to nucleotides. The distribution of thymine-derived bases 5-*p*hmdUMP and 5hmdUMP was estimated at ~20% and ~80%, respectively (i.e. not all 5hmdUMP in the genome can be phosphorylated, suggesting certain sequence specificity for phosphorylation).

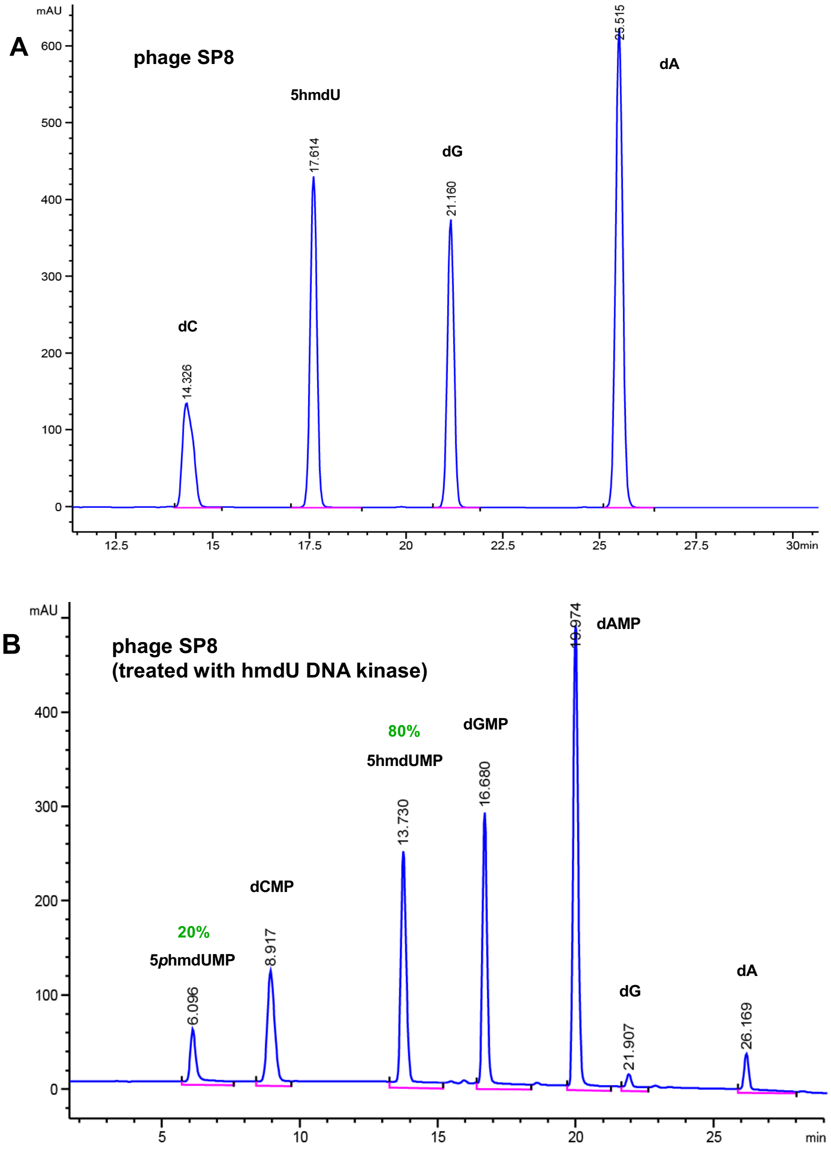

Supplement: Supplementary file 1 [file Data_Sheet_1.PDF]
